# Current treatment of oral candidiasis: A literature review

**DOI:** 10.4317/jced.51798

**Published:** 2014-12-01

**Authors:** Carla Garcia-Cuesta, Maria-Gracia Sarrion-Pérez, Jose V. Bagán

**Affiliations:** 1 Dentist. Postgraduate in Oral Medicine; 2 Associate profesor of Oral Medicine Unit. Department of Stomatology. University of Valencia; 3 Chairman of Oral Medicine. Oral Medicine Unit. Department of Stomatology. University of Valencia. Head of the Department of Stomatology and Maxilofacial Surgery. Valencia University General Hospital

## Abstract

Candidiasis or oral candidosis is one of the most common human opportunistic fungal infections of the oral cavity. This pathology has a wide variety of treatment which has been studied until these days. The present study offers a literature review on the treatment of oral candidiasis, with the purpose of establish which treatment is the most suitable in each case. Searching the 24 latest articles about treatment of candidiasis it concluded that the incidence depends on the type of the candidiasis and the virulence of the infection. Although nystatin and amphotericin b were the most drugs used locally, fluconazole oral suspension is proving to be a very effective drug in the treatment of oral candidiasis. Fluconazole was found to be the drug of choice as a systemic treatment of oral candidiasis. Due to its good antifungal properties, its high acceptance of the patient and its efficacy compared with other antifungal drugs. But this drug is not always effective, so we need to evaluate and distinguish others like itraconazole or ketoconazole, in that cases when Candida strains resist to fluconazole.

** Key words:**Candidiasis, treatment, miconazole, fluconazole, nystatin.

## Introduction

The incidence of fungal infections has been increasing over the last decades, being more prevalent in developed countries (1). An increase incidence of the infections is associated with some predisposing factors ( Table 1) as the use of dentures, xerostomia, prolonged therapy with antibiotics, local trauma, malnutrition, endocrine disorders, increased longevity of people, among other states that diminish the quality of defense of the individual (2). Oral candidiasis is one of the most common clinical features of those patients infected with the human immunodeficiency virus [HIV], this manifestation was seen in up to 90% of individuals infected with HIV (3).

**Table 1:**
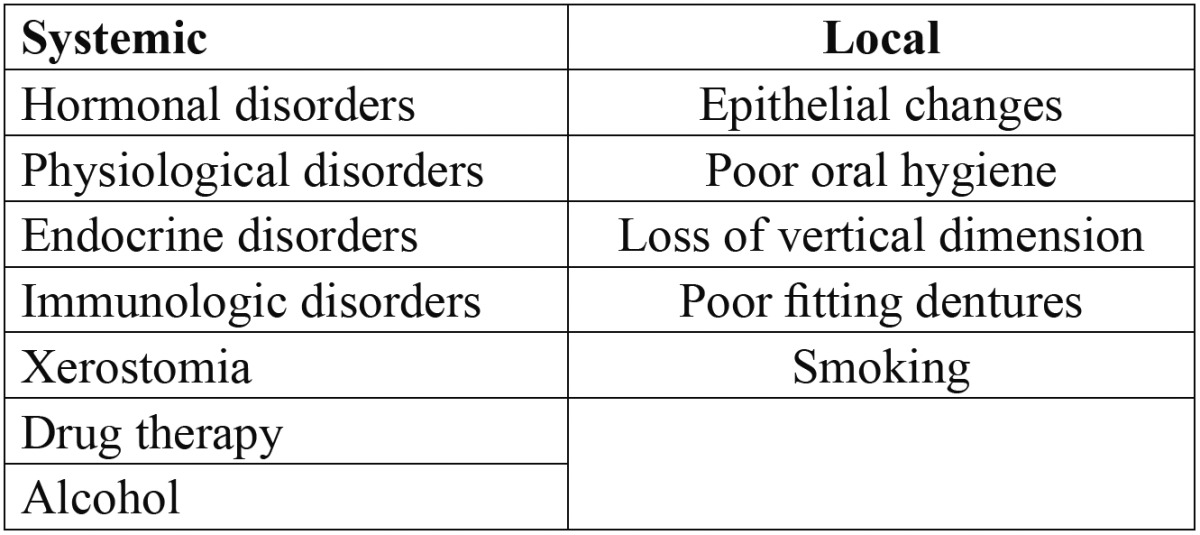
Predisposing factors.

Oropharyngeal candidiasis is caused by the genus *Candida*; it is possible to isolate about 150 species. Many of these remain as a commensal micro-organism in humans, which could act as an opportunistic pathogens often associated with predisposing factors attributed to the organism, thereby causing acute or chronic infections (4). The most important of these species is *C. albicans*, which is most commonly isolated from the oral cavity and is believed to be more virulent in humans, occurring in approximately 50% of the cases of candidiasis.

Clinically there are a number of different types of oral candidiasis ( Table 2). Therefore the choice of therapy is guided by the type of candidiasis.

**Table 2:**
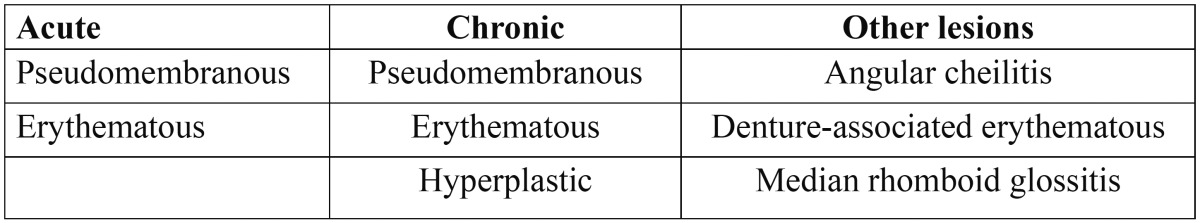
Clinical classification.

The diagnosis of oral candidiasis is essentially clinical and is based on the recognition of the lesions by the professional, which can be confirmed by the microscopic identification of *Candida* (5). The techniques available for the isolation of *Candida* in the oral cavity include direct examination or cytological smear, culture of microorganisms and biopsy which is indicated for cases of hiperplasic candidiasis because this type could present dysplasias (6).

The treatment of oral candidiasis is based on four fundaments (7): making an early and accurate diagnosis of the infection; Correcting the predisposing factors or underlying diseases; Evaluating the type of *Candida* infection; Appropriate use of antifungal drugs, evaluating the efficacy / toxicity ratio in each case.

When choosing between some treatments it will take into account the type of *Candida*, its clinical pathology and if it is enough with a topical treatment or requires a more complex systemic type (8), always evaluating the ratio efficacy and toxicity (9). The different drugs are contained in  Table 3.

**Table 3:**
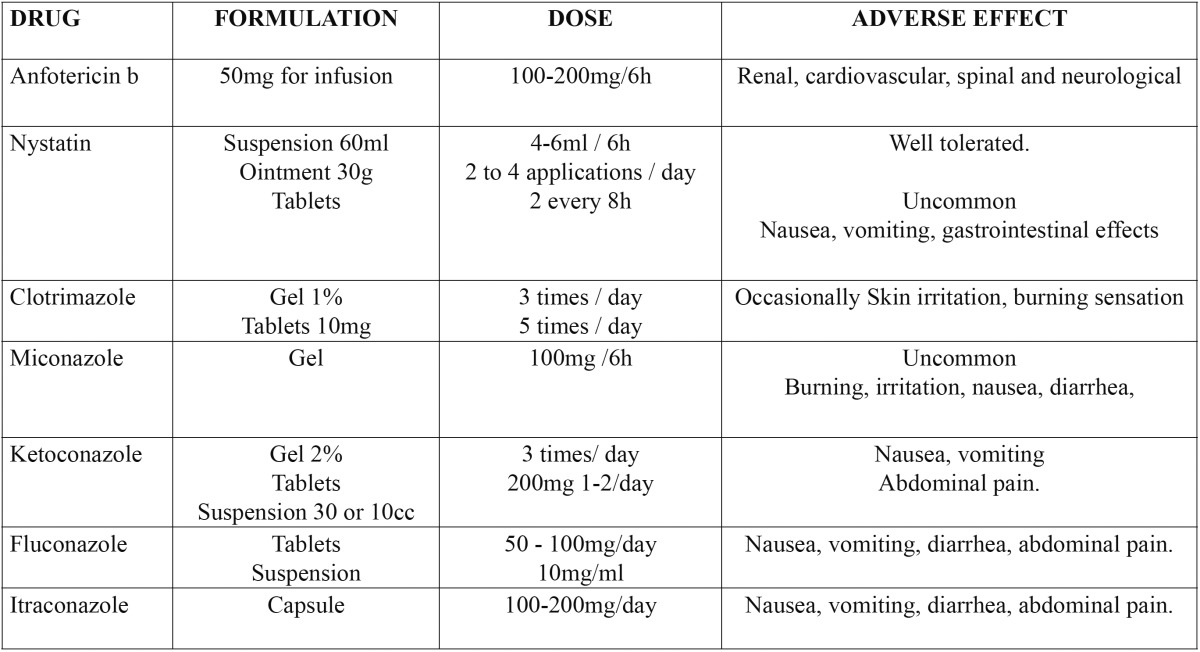
Antifungal agents. Vademecum.

Regular oral and dental hygiene with periodic oral examination will prevent most cases of oral candidiasis, so it is need to make the patient aware of oral hygiene measures. Oral hygiene involves cleaning the teeth, buccal cavity, tongue, and dentures. As well as the use of anti- *Candida* rinses such as Chlorhexidine or Hexetidine, so that they can penetrate those areas where the brush does not. In addition, the need to remove the dentures at night and wash it consciously, leaving it submerged in a disinfectant solution like Chlorhexidine (10).

This study provides a literature review of the treatment of oral candidiasis and its objectives are to establish general guidelines for treatment of oral candidiasis; Assess the drug of choice for local treatment of oral candidiasis; Assess the systemic treatment for oral candidiasis.

## Material and Methods

A Medline-PubMed search was made using the following key words: “ oral candidiasis” OR “oral candidosis” AND amphotericin, “oral candidiasis” OR “oral candidosis” AND nystatine, “oral candidiasis” OR “oral candidosis” AND miconazole, “oral candidiasis” OR “oral candidosis” AND ketoconazole, “oral candidiasis” OR “oral candidosis” AND clotrimazole, “oral candidiasis” OR “oral candidosis” AND fluconazole, “oral candidia-sis” OR “oral candidosis” AND itraconazole, “oral candidiasis” OR “oral candidosis” AND treatment, “oral candidiasis” OR “oral candidosis” AND “antifungal therapy”.The key words were validated by the MeSH [Me-dical Subject Headings] dictionary, with use of the boolean operator “AND” to relate them.

The following limits for inclusion of the studies were established: articles published from 2000, publications in English and Spanish and publications of studies in humans. All systematic reviews, clinical trials, meta-analysis and comparative studies were considered in this review.

A total of 109 articles were identified, of which 30 were selected after reading the abstracts. Following analysis of the 30 articles, we finally included a total of 24, since those publications that did not fit the aims of the present study were excluded.

## Results

A total of 24 articles were found about antifungal treatment, of which 20 were clinical trials, 3 systematic re-views and 1 a clinical case ( Table 4,  Table 4 (Cont)).

**Table 4:**
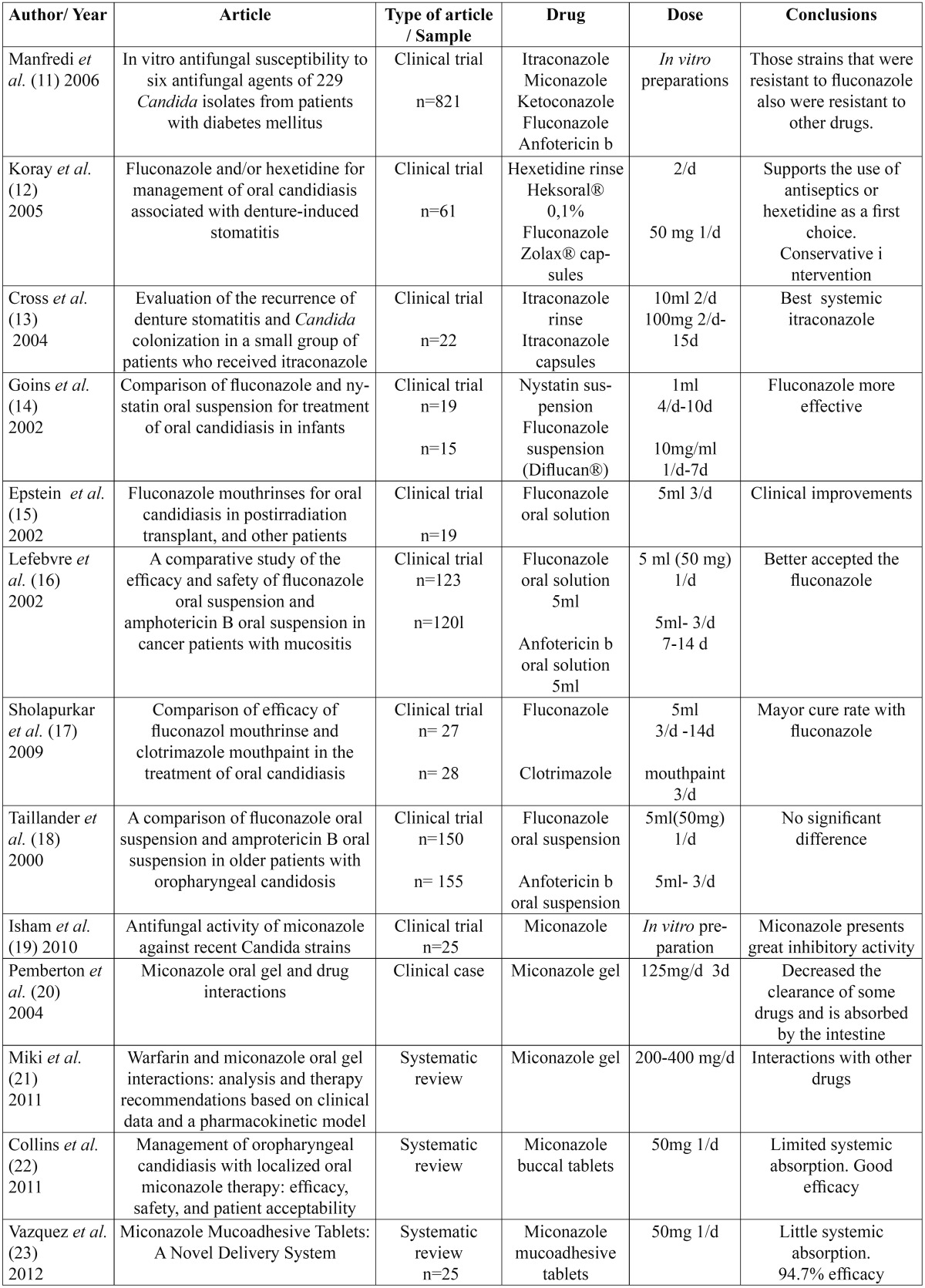
Summarized articles.

**Table 4 (Cont):**
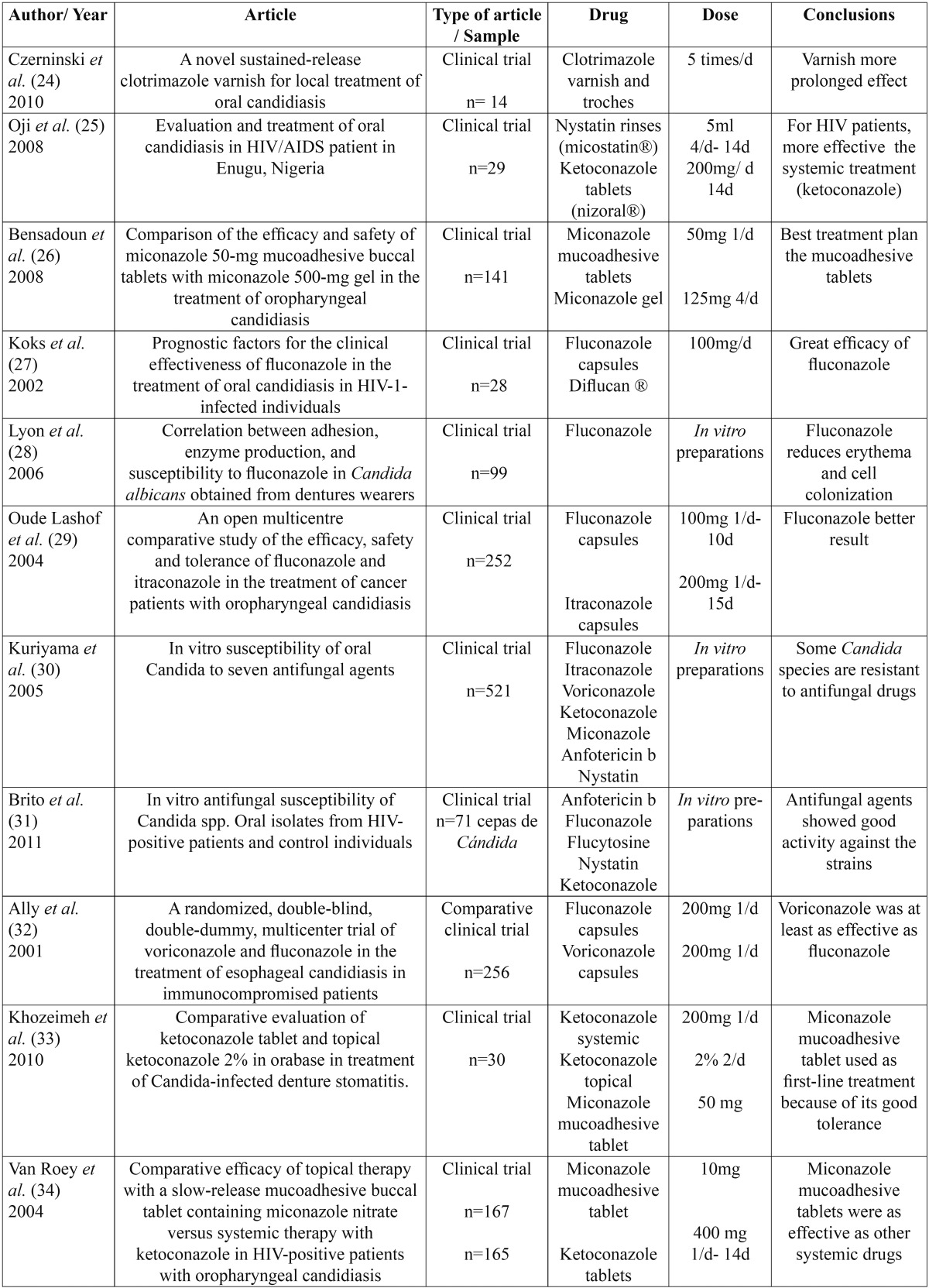
Summarized articles.

## Discussion

Candida infection today is highly prevalent, especially the increase in carriers of removable dentures and poor oral hygiene society. Depending on its virulence, location and type of candidiasis there will carry on one treatment or another.

First has been supported the use of conservative measures before starting drug treatment, promoting good oral hygiene along with removing the dentures at night, thereby it will benefit the removal of the biofilm layer generated in the prosthetic surface (11). Dentists should also correct the predisposing factors and underlying diseases and try to promote the use of oral antiseptic and antibacterial rinses such as Chlorhexidine or Hexetidine (12). These measures are very effective in patients with denture stomatitis (12). It was also found in the study of Cross et al. (13) that in patients with good oral hygiene the recurrence of candidiasis after 3 years was lower.

Regarding the pharmacological treatment of candidiasis can be distinguished between two procedures. Topical drugs, which are applied to the affected area and treat superficial infections and systemic drugs those that are prescribed when the infection is more widespread and has not been enough with the topical therapy.

As first choice for local treatment has been for years the nystatin at doses of 100 000 IU/ml [5ml 4 times daily] and amphotericin b at 50mg [5ml 3 times per day]. This choice is because they are poorly absorbed by the intestinal tract and therefore most of the antifungal is excreted without undergoing any change, thereby reducing hepatotoxicity (14). However, the unpleasant taste and prolonged pattern compromise treatment compliance by the patient (14-16).

Throughout the years it has been studying the effectiveness of other drugs like fluconazole oral solution. Many authors have focused on evaluating the efficacy and safety of fluconazole oral solution for the treatment of oropharyngeal candidiasis, especially pseudomembranous type, giving good results, although many studies are still needed (14-18).

In a recent study conducted in 19 patients with pseudomembranous candidiasis show that fluconazole suspension in distilled water [2mg/ml] reaches a 95% cure.

The guideline was to rinse with 5ml of the drug solution for 1 minute and then spit it out and repeat this action 3 times a day for 1 week. Another study which included 36 children with pseudomembranous candidiasis showed that fluconazole oral suspension 10mg/ml dose gave better results than nystatin. The main problem was the poor adherence of the nystatin to the oral mucosa and thus the quick ingestion of the suspension, resulting in a lower efficiency (14).

On the other hand, in another study comparing amphotericin b suspension, the fluconazole oral suspension gave better results in terms of the eradication of *Candida* (16). The same was corroborated by Taillandier *et al.* (18), which reported that fluconazole oral suspension was as effective as amphotericin b, but it was better accepted by the patient.

Fluconazole oral suspension is administered in a dosage of 10 mg / ml aqueous suspension by administering 5 ml daily for 7 or 14 days. Different studies show that it is a very effective drug against pseudomembranous candidiasis, as it has good adhesion to the surface of the oral mucosa and a rapid symptomatic response. It also offers the convenience of a one-daily dosing, which may explain the better patient compliance (14-18).

Another topic drug widely used is miconazole (19). We found it in the form of gel, applying it directly on the affected area, at doses of 200-500 mg per day, divided into 4 times. Despite its good properties it has the draw-back of possible interaction with other drugs, such as warfarin. This is because the antifungal inhibit the enzyme cytochrome P-450, which affects the clearance of certain drugs (20,21). In addition, this drug is absorbed by the intestine, therefore care must be taken when is administrated.

It has been introduced in the market an alternative presentation of miconazole. A one-daily miconazole 50 mg mucoadhesive buccal tablet. It has a limited systemic absorption. Its performance is mostly local and it has a convenient application form. Patients are instructed to apply the rounded side of the 50 mg tablet to the upper gum region just above the right or left incisor following brushing of teeth in the morning. The tablet should be held in place until dissolved (22,23). It has the advantage of being applied once daily instead 5 times a day with clotrimazole (24), and 4 times daily with nystatin (25).

It has been demonstrated the effectiveness of this new form of administration in the study of Bensadoun *et al.* (26). 141 patients with head and neck cancer with clinical signs and symptoms of oropharyngeal candidiasis received 50 mg mucoadhesive tablets of miconazole daily or 125 mg miconazole gel four times per day. Clinical improvement was not significant between the two groups, but the mucoadhesive tablets exhibited higher salivary concentrations and better tolerance for the patient. Despite being more expensive, offers an effective, safe, and well tolerated topical treatment for oropharyngeal candidiasis (22,23,26).

- Systemic treatment:

In spite of knowing the efficacy of the drugs listed above, when it comes to a more generalized candidiasis or immunocompromised patients, these would not be sufficient. For those cases would have to resort to treatment with systemic drugs (25).

Since its introduction, fluconazole has been used to treat systemic *Candida* infections because of its efficacy and good tolerability. The appropriate dose is between 50-100 mg daily (27). Furthermore, when dealing with immunocompromised patients, such as those HIV-infected, or cancerous, this drug has good effects doubling the dose (28,29). Its efficacy has been demonstrated (27). The dose was individualized depending on the severity and type of candidiasis. Patients with pseudomembranous type started with 100 mg fluconazole daily; patients with erythematous variety started with 50 mg fluconazole. Therefore, according to the clinic and the virulence of the infection the dose would be titrated, giving good results, and increasing the guideline in those cases where the fungal infection did not decrease (27).

To support the efficacy of this drug it has been compared with other systemic antifungal agents (29). In one randomized study, the efficacy of fluconazole [100mg per day for 10 days] and itraconazole [200mg per day for 15 days] was compared in patients with oropharyngeal candidiasis. The results were a clinical and mycological improvement of 66% for the first group and 54% for those treated with itraconazole. The main conclusion of this study is that in patients with oropharyngeal candidiasis, fluconazole has a significantly better clinical and mycological cure rate compared with itraconazole. The failures of itraconazole may be explained by drug interactions and the unpredictable absorption of itraconazole capsules. But when fluconazole failed, itraconazole was prescribed to these patients, having good results. So it is said that it was a good drug for fluconazole-resistant Candida strains (29).

As it has been suggested above, it may happen that the *Candida* strains were not susceptible to fluconazole, and it has not any effect. In that case it will be used other drugs like itraconazole or newest ones as voriconazole (30). Keeping always in mind that strains which were resistant to fluconazole were also resistant to other drugs (31).

The new triazol antifungal voriconazole [200 mg per day] has been shown to be a potent drug. Ally *et al.* (32) compared the efficacy of voriconazole and fluconazole in the treatment of esophageal candidiasis. The success rate was 98.3% for voriconazole and 95.1% for fluconazole. The results show clearly that voriconazole is at least as effective as fluconazole in the treatment of candidiasis. It suggests that this new agent may be a useful alternative for fluconazole-resistant *Candida* strains (32). Because of being a new there are little strains resistant to voriconazole. The voriconazole has an important role in the treatment of candidiasis (30), although it is still not fully established in the market, so many more studies and research would be needed.

There have been several studies comparing topical and systemic drugs. In a study to treat denture stomatitis have been compared the use of ketoconazole tablets [200mg daily] with topical ketoconazole [2% twice daily] and miconazole mucoadhesive tablets (33). Due to the adverse effects of ketoconazole (31) like nausea, vomiting and gastrointestinal problems it has been supported the use of other drugs when treating prosthetic candidiasis (34). Thus the use of miconazole mucoadhesive tablet was established as the drug of first line of defense for this type of candidiasis.

General treatment guidelines include after the completion of an early diagnosis, the correction of predisposing factors or underlying diseases and maintaining a good oral hygiene. Moreover using antiseptic agents such as Chlorhexidine or Hexetidine, as well as removing dentures at night. All of that in order to obtain well results in the treatment of oral candidiasis as first line of defense, continuing the application of antifungal drugs. Beginning with local treatment and keeping up the systemic ones for those patients who do not respond to topical treatment or in immunocompromised patients.

It has recently been found that fluconazole oral suspension as a local treatment, at a dose of 2 mg/ml 3 times daily or 10 mg /ml once daily, gives good clinical results, besides the better patient compliance due to the dosage and its pleasant taste. Despite not being currently the most widely used locally because it requires further clinical studies. Nowadays the most used drugs remains in nystatin solution which contain 100 000 IU / ml [5ml 4 times daily] and miconazole gel [200 to 500 mg per day divided into 4 doses]. Moreover miconazole mucoadhesive tablets [50 mg once daily] which are considered effective in the treatment of oropharyngeal candidiasis, but their high cost is one of the main problems.

Fluconazole at doses between 50-100 mg per day is the systemic drug of choice because it has high efficacy and tolerability by the patient. However it is important to think about the voriconazole which is as effective as fluconazole but is still under study. Also it is need to know about other drugs such as itraconazole, which are effective when *Candida* strains are resistant to fluconazole.
